# Near-complete genome of two genotype II African swine fever viruses recovered from domestic pigs in Tanzania

**DOI:** 10.1128/mra.00635-25

**Published:** 2025-10-24

**Authors:** Jean N. Hakizimana, Clara Yona, Charles Kayuki, Mariam R. Makange, Ester K. Adamson, Amanda Warr, Christine Tait-Burkard, Hans J. Nauwynck, Gerald Misinzo

**Affiliations:** 1 Oliver R. Tambo Africa Research Chair for Viral Epidemics, SACIDS Foundation for One Health, Sokoine University of Agriculture 108091, , Morogoro, Tanzania; 2 SACIDS Africa Centre of Excellence for Infectious Diseases, SACIDS Foundation for One Health, Sokoine University of Agriculture 108091, , Morogoro, Tanzania; 3 Department of Biosciences, Solomon Mahlangu College of Natural and Applied Sciences, Sokoine University of Agriculture 108091, , Morogoro, Tanzania; 4 Oxford Nanopore Technologies 120806, Oxford, United Kingdom; 5 The Roslin Institute and Royal (Dick) School of Veterinary Studies, University of Edinburgh 70677 , Midlothian, United Kingdom; 6 Laboratory of Virology, Faculty of Veterinary Medicine, Ghent University 366760, , Merelbeke, Belgium; 7 Department of Microbiology, Parasitology and Biotechnology, College of Veterinary Medicine and Biomedical Sciences, Sokoine University of Agriculture 108091, , Morogoro, Tanzania; Katholieke Universiteit Leuven, Leuven, Belgium

**Keywords:** African swine fever virus, complete genome sequencing, Oxford nanopore, Tanzania

## Abstract

Two near-complete genomes of genotype II African swine fever viruses (ASFV) recovered from domestic pigs in Mbozi district, Tanzania, in 2017 were generated using tiled amplicon Oxford nanopore sequencing. These two ASFV genomes described in this study were closely related to other genotype II isolates reported worldwide.

## ANNOUNCEMENT

African swine fever (ASF) is a transboundary animal disease with a mortality rate reaching 100% (1). Whole genome sequencing of ASF virus (ASFV) provides insights for the design of control strategies. The ASFV is an enveloped, double-stranded DNA virus belonging to the family *Asfarviridae*, genus *Asfivirus* (2). To overcome ASFV sequencing challenges, including the dominance of host DNA, a tiled amplicon sequencing approach has been developed (3, 4).

Tissue samples, including spleen and mesenteric lymph nodes, collected during the 2017 ASF outbreak in Mbozi district in southern Tanzania (ASFV/TAN/17/Mbozi/1 and ASFV/TAN/17/Mbozi/2) were tested for ASFV and clustered within ASFV genotype II as previously described (5). The DNA was extracted using the QIAamp DNA purification kit (Qiagen, Hilden, Germany), followed by polymerase chain reaction (PCR) amplification as previously described (3). A total of 32 tiled primer pairs were used to amplify fragments of about 7 kb with overlaps of 1 kb between adjacent amplicons using PCR Bio VeriFi Hot Start high-fidelity DNA polymerase. Amplicon size was verified by 1% agarose gel electrophoresis (Gel DocTM EZ Imager, Bio-Rad, Hercules, CA). The amplicons were pooled for library preparation using the ligation sequencing kit (SQK-LSK109, Oxford Nanopore Technologies, Oxford, UK) and sequenced on an R9.4.1 flow cell using MinION MK1c with basecalling and demultiplexing performed by Guppy v5.0.14 (Oxford Nanopore Technologies, Oxford, UK). The Lilo pipeline (https://github.com/amandawarr/Lilo) was used for data analysis. Lilo uses a reference to sort the amplicons and separate reads into amplicons by alignment position using bedtools v2.30.0, followed by polishing against the highest quality reads, while primer sequences are removed using Porechop v0.2.3, and assembly is performed with Scaffold_builder v2.3. In this study, the Lilo pipeline was modified due to some misassembly caused by chimeric amplicons in the sequencing data. In the rule “assign”, bedtools intersect command parameters were edited to include “-F 0.85 f 0.85” to limit the selection to sequences that contained only the target amplicon, while allowing flexibility for real indels. The resulting assemblies were evaluated using the Quality Assessment Tool (QUAST) version 5.0.2 (6).

**Fig 1:**
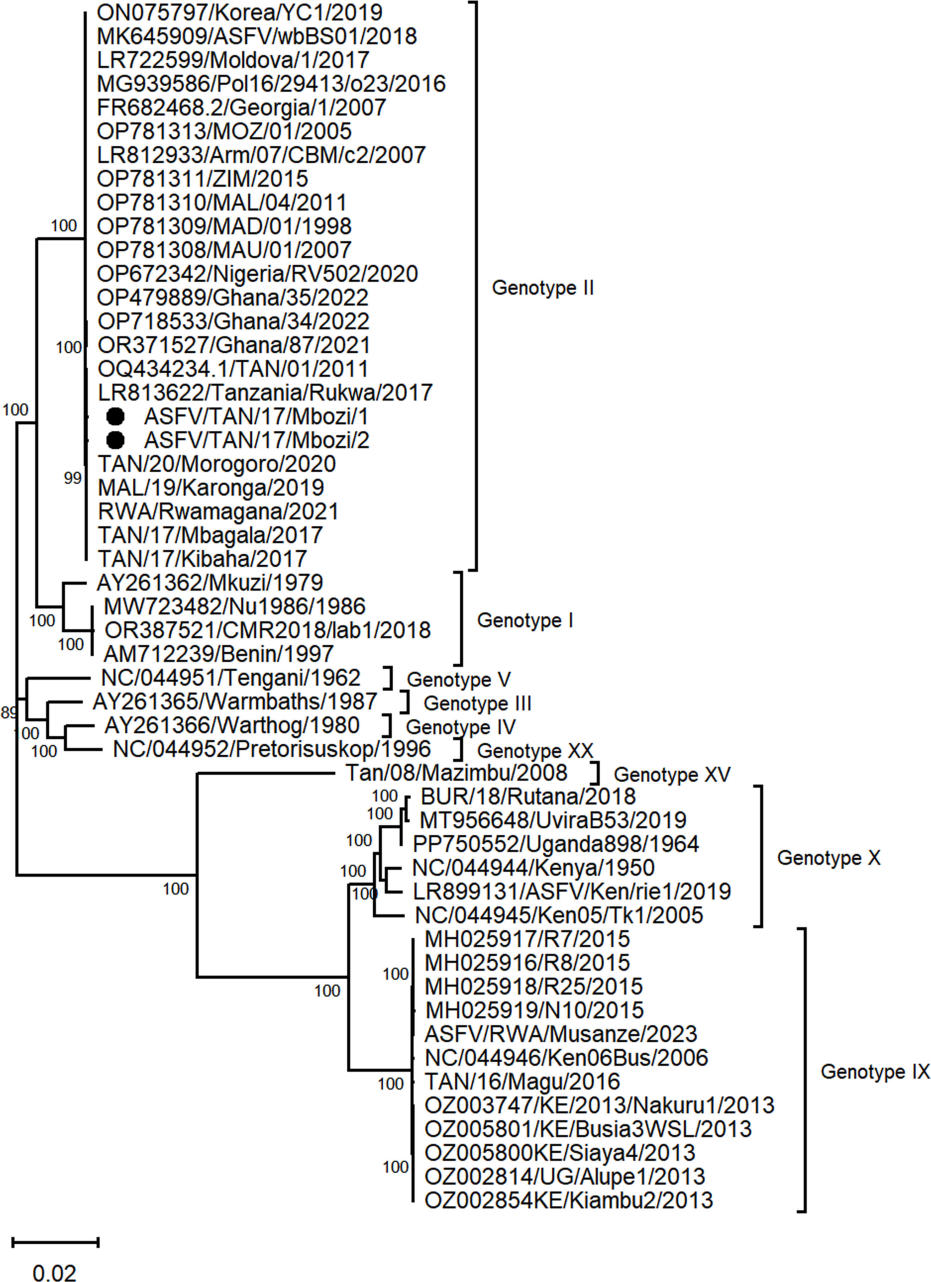
Maximum Likelihood phylogenetic tree reconstructed using ASFV complete genome nucleotide sequences including those described in this study (marked by a black circle) and those previously reported worldwide available at the NCBI GenBank. The phylogeny was inferred using Tamura (992) nucleotide substitution model, as determined by the Bayesian Information Criterion model selection analysis. The sequences were aligned using MAFFT version 7.221 and phylogenetic tree reconstructed using 1,000 bootstrap replications as implemented in MEGA 12. The scale bar indicates nucleotide substitution per site and the node values show the percentage of bootstrap support.

The nucleotide sequences of the assembled ASFV strains had a genome size of 172,585 and 167,022 base pairs (bp), average coverage of 2,500 and 4,370 reads per nucleotide, and a GC content of 38.95 and 39.17% for the strains ASFV/TAN/17/Mbozi/1 and ASFV/TAN/17/Mbozi/2, respectively (Table 1). After the NCBI GenBank database search and phylogenetic reconstruction (Fig. 1), the sequences described in this study were closely related to the TAN/01/2011 ASFV isolate (OQ434234) (7) belonging to genotype II with a nucleotide identity of 99.82% and a query coverage of 100%. In addition to the lack of the highly repetitive 3′- and 5′ telomeric regions for the two sequences, indels were observed along the alignment after comparison with the ASFV genotype II reference genome Georgia2007/1 (FR682468.2), including a deletion of fragments of 5,465 and 5,220 bp at positions 16,190–21,665 and 169,120–174,340, respectively, leading to the truncation of the MGF 360-1Lb CDS and ASFV G ACD 01980 CDS in both ASFV strains described in this study.

**TABLE 1:** Summary of the basic statistics of the sequencing results described in this study

Isolate ID	Total number of reads	ASFV specific reads	N50	Percentage of ASFV specific reads (%)	Mean phred quality score	Assembled ASFV genome size (bp)	Mean genome coverage depth	GC content (%)	Accession number
ASFV/TAN/17/Mbozi/1	245,015	212,621	6,930	86.78	11.5	172,585	2,500	38.95	PV740684
ASFV/TAN/17/Mbozi/2	482,432	419,948	6,031	87.056	11.7	167,022	4,370	39.17	PV740683

## Data Availability

The near complete nucleotide sequences generated in this study are available at NCBI GenBank under accession numbers PV740684 and PV740683. The raw data of the nucleotide sequences described in this paper have been deposited at the NCBI Sequence Read Archive (SRA) with BioProject accession number PRJNA1274503.
